# In cats with cranial cruciate rupture are better long-term outcomes achieved by surgical or conservative management?

**DOI:** 10.18849/ve.v10i2.696

**Published:** 2025-04-28

**Authors:** Philip Montgomery

**Keywords:** stress

## Abstract

**PICO question:**

In cats diagnosed with isolated rupture of the cranial cruciate ligament, does surgical intervention result in improved long-term function, when compared with conservative management?

**Clinical bottom line:**

**Category of research:**

Treatment.

**Number and type of study designs reviewed:**

One retrospective cohort study comparing surgical management and conservative management.

**Strength of evidence:**

Weak.

**Outcomes reported:**

This study evaluated and compared the long-term Feline Musculoskeletal Pain Index (FMPI) of patients with a cranial cruciate injury (39–87 months post injury). Patients that were managed conservatively showed statistically significant (P = 0.017) lower FMPI scores long term.

**Conclusion:**

Conservative management can be considered in cats presenting with isolated cranial cruciate rupture; however, further research is required to establish the optimum treatment in these cases. Given the limitations of this study and the current lack of evidence, clinicians must rely on their clinical judgement at this point when managing this condition until further research is available.

## How to apply this evidence in practice

The application of evidence into practice should take into account multiple factors, not limited to: individual clinical expertise, patient’s circumstances and owners’ values, country, location or clinic where you work, the individual case in front of you, the availability of therapies and resources.

Knowledge Summaries are a resource to help reinforce or inform decision-making. They do not override the responsibility or judgement of the practitioner to do what is best for the animal in their care.

## The evidence

There is weak evidence provided by Boge et al. (2020) that cats with isolated cranial cruciate ligament rupture managed conservatively may experience less chronic pain and stifle dysfunction than those managed surgically with a lateral tibiofabellar suture. There is no published literature assessing the long term outcomes of any other surgical techniques for the treatment of feline cranial cranial cruciate ligament rupture.

## Summary of the evidence

**Boge et al. (2020):** Cranial cruciate ligament disease in cats: an epidemiological retrospective study of 50 cats (2011–2016) **Aim: **To compare the long term outcomes of surgical and non-surgical management of feline cranial cruciate ligament disease using a Feline Musculoskeletal Pain Score questionaire completed by patient owners.

Population	Cats of both sexes and several breeds with cranial cruciate ligament disease diagnosed at two university hospitals (Swedish University of Agricultural Sciences and Norwegian University of Life Sciences) in a six year period between January 2011 and December 2016.
Sample Size	50 cats.
Intervention Details	Two groups of cats with cranial cruciate rupture: 22/50 cats (44%) were managed surgically with a lateral fabellotibial suture. 12 nylon suture, 2 polypropylene, 7 polyethylene, 1 polyester. 19/22 cases had an arthrotomy performed. In all 19 cases a complete cranial cruciate ligament rupture was identified. 9/19 cases that had an arthrotomy had a meniscal injury reported. In 5/22 surgically managed cases, there was a multiligament injury to the stifle. In 4 cases there was damage to the collateral ligament. In 3 cases, there was injury to both the caudal and cranial cruciate ligaments. 18/ 22 surgical cases received antibiotic treatment, 11 of which were perioperative only, 7 perioperative and postoperative. 6/22 surgical cases had a postoperative complication and 3 of which underwent a second operation. 28/50 cats (56%) were managed conservatively. The nature of this conservative management was not defined.
Study design	Retrospective cohort study.
Outcome studied	Feline Musculoskeletal Pain Index (FMPI) long term (39–87 months post injury). This is a validated mobility questionnaire consisting of 18 questions about the ability of the patient to perform different activities that was completed by the owners. A score of -1 indicated an above normal ability, a score of 4 indicated not able to perform the activity at all. The questionnaire also contained 2 pain questions, regarding pain over the last week and pain over the last day, these were scored between 0 (no pain) and 3 (severe pain). A final question of overall quality of life was scored from 0 (excellent) to 3 (poor). A low score indicates less chronic pain (P = 0.04), a total score of 3 is regarded as the cut off for normal cats. The maximum possible score is 81.
Main findings (relevant to PICO question)	24 owners completed the FMPI scoring questionnaire, 12 from the surgically managed group and 12 from the conservatively managed group. Cats with cranial cruciate disease managed conservatively showed a statistically significant (P = 0.017) lower FMPI score long-term (median FMPI score 0.5, range -6–7) when compared with those managed surgically (median FMPI score: 5, range 0–15).
Limitations	The study population in the participating centers may not represent the general population. This is a small study with a low number of cases—22 surgically managed cats, with only 17 with an isolated cranial cruciate ligament rupture. The retrospective nature of the study means there will have been variation in treatment and management recommendations, especially between clinicians. Complete or partial cranial cruciate ligament rupture was not defined, all cases managed surgically with arthrotomy had a complete rupture. The degree of ligament rupture was unknown in conservatively managed cases and so some of the cases managed conservatively may only have partial rupture of the cranial cruciate ligament, giving more stifle stability and a different outcome due to this. The outcome studied (FMPI score) is based on owner survey: lameness and pain in cats is challenging to assess accurately. The complication rate was not defined. Some reported post operative complications occurred a long period of time after the surgery (up to 532 days post operatively). There is potential that new injuries/traumas to the stifle contributed to these complications. Complications can vary with surgical technique and experience, in this study there was no statistically significant difference in the frequency of complications between the two clinics (P = 0.35) or between cases with multiligament injury and those with isolated cranial cruciate disease (P = 0.59). The complication rate of conservatively managed cases is not defined, it is not reported if these cases had ongoing or recurrent episodes of lameness or future injuries. Severity of instability/lameness may be a factor in treatment decision: no information on degree of lameness at presentation was available. Concurrent injuries or health issues may impact the healing process of the stifle injury this includes trauma to other areas (e.g. femoral fracture) or comorbidities (e.g. Feline leaukemia virus). Patients with concurrent injuries, e.g. femoral facture, may be more inclined to be managed surgically if surgery was already required for the other injuries. Other factors may have affected the decision of whether to manage a case surgically or conservatively, including, encouragement of the examining veterinary surgeon towards a particular treatment which can be due to logistical, financial reasons, or personal opinions regarding cruciate management. Owners may also have their own inherent bias for or against surgical management which would change this decision. Bilateral cruciate disease was present in 7 cats, this may have affected the healing process and thus the long-term outcome. Unknown presence of meniscal injury in conservatively managed population. Meniscal injury has been shown to significantly impact outcome. Patients with meniscal injury may be lamer on presentation and, therefore, could be more likely to be surgically managed. Variation in surgeons: 12 different surgeons in total contributed cases with different levels of experience with this surgery, surgeon qualification and time in practice is not clarified. No diagnostic imaging was performed: diagnosis of cranial cruciate ligament rupture was based on physical exam only. Conservative treatment was used more often at the Swedish University of Agricultural Sciences. This could suggest a preference for this method among veterinary staff at this center, or another factor impacting the decision-making process. No osteoarthritis grading was performed to assess for underlying or pre-existing joint pathology. 5/28 cats managed surgically had multiligament injuries and not isolated cranial cruciate injuries—while the FMPI scores are still significantly higher in surgical cases when these cases are excluded. This reduces the case numbers further. Bias of owner assessment cannot be excluded. For example, owners that opt for surgery, may be more vigilant of signs of discomfort. No power analysis was reported. Potential for responder bias, owners may be more likely to respond if persistent lameness is noted. Potential for non-responder bias as not all owners completed the FMPI questionnaire—FMPI questionnaires were completed for 24/50 cases in total. There was no clinical assessment of the patient’s long term function, owner assessment of the patients was relied on entirely.

Table note...

## Appraisal, application and reflection

Cranial cruciate disease is an area of active research in veterinary literature due to how frequently cranial cruciate disease occurs in the canine population (Taylor-Brown et al., 2015). Two distinct pathogenesis of cranial cruciate disease in dogs have been proposed: acute traumatic rupture and chronic degeneration (Griffon, 2010). Canine cruciate disease can be managed conservatively or through a range of surgical procedures. Surgical management has been shown to be superior to conservative management in both short- and long-term outcomes in canine patients, based on force plate analysis (Wucherer et al., 2013). Conservative management is still a popular option in small breed dogs (< 15kg) (Comerford et al., 2013), as historically a successful outcome was reported in 75-90% of cases. (Pond & Campbell, 1972; Vasseur, 1984), however, in Pond & Campbell (1972), which reported a 90% success rate, this success was based on owner report only, with no objective measurement of lameness performed and so must be interpreted cautiously. Vasseur (1984), reported a successful outcome in only 75% of patients, and showed that this recovery was often prolonged, taking 4 months on average. Of the patients that had a reported resolution of lameness, there was evidence of muscle atrophy in 19% of cases, an increased medial buttress in 67%, 43% had an instability on cranial drawer testing and 100% showed evidence of progression of osteoarthritis (Vasseur, (1984); Brioschi & Arthurs, (2021)). More recently, Kwananocha et al. (2024) demonstrated a similar short-term (12 week) improvement in orthopaedic assessment score, when comparing cases managed conservatively and those managed surgically with tibial plateau levelling Osteotomy (TPLO) in dogs weighing less than 10 kg. However, the TPLO group demonstrated an increase in thigh muscle circumference on the affected leg at 70 days postoperatively that was not demonstrated in the conservatively managed group. It is worth noting that this was a small study, with a short follow-up, significant variation between cases, no force plate analysis, and limited gait assessment, so the similarity in short term outcome must be interpreted very carefully.

Several surgical techniques are used regularly in the treatment of canine cruciate disease, including osteotomy surgeries and techniques that provide extracapsular support (Bergh et al., 2014). The (TPLO) is one of the most commonly reported osteotomy procedures (Bergh et al., 2014), and has been shown to have superior outcomes when compared to lateral fabellotibial suture in canine patients (Gordon-Evans et al., 2013).

The treatment options and outcomes when treating feline cranial cruciate disease are not as well understood as canine cranial cruciate disease due to the lack of published evidence on this topic. The aetiopathogenesis of isolated feline cruciate disease remains unclear; however, a histopathology study of rupture feline cruciate ligaments found no evidence of degeneration (Wessely et al., 2017). Feline cruciate ligament rupture is also reported as part of multiligament traumatic injury to the stifle (Coppola et al., 2021). Multiligament stifle injuries are outside the scope of this Knowledge Summary, which focuses purely on isolated cranial cruciate disease. As in the canine patient, feline cranial cruciate rupture can be treated conservatively, or surgically. Conservative management of feline cruciate disease has been shown as a viable option for management of feline cruciate disease (Stoneburner et al., 2022), with 15/18 (83%) cats reportedly achieving clinical normality within 3 months. The most reported surgical technique for the management of feline cruciate disease is the lateral fabellotibial suture (Harasen, 2005). There are reports of osteotomy procedures in the treatment of feline cranial cruciate ruptures (Minder et al., 2016); however, there are limited case numbers, with no long-term follow-up. Muscle transposition techniques have also been described, transposition of the biceps femoris muscle has been reported in cats with reportedly successful results, however, no follow up on outcomes beyond 90 days postsurgery are available at this time (Sen, 2019).

The evidence for long term outcomes of the different management options for feline cranial cruciate rupture is limited. Cats with previous cranial cruciate disease have been shown to have a long-term gait abnormality (Stadig et al., 2016). In this study six of the 10 cats were managed surgically, and four of the 10 cats were managed conservatively. There was no significant difference between the groups and all cats showed a long-term gait abnormality and behavioral changes.

The only published paper that compares the long-term outcomes between cases managed with surgery to those managed conservatively, reported a lower long-term Feline Musculoskeletal Pain Index (FMPI) score in the conservatively managed cases (39–87 months post injury) (Boge et al., 2020)). However, it is important to recognise that this is a small, retrospective study, with an owner-assessed outcome.

The FMPI score has been evaluated and found to have sound reliability, internal consistency, and good discriminatory ability (Stadig et al., 2019). As this is a general questionnaire of all aspects of the patient’s activity, there is potential that comorbidities contributed to patient scores. As no diagnostic imaging or osteoarthritis scoring was performed in this study, pre-existing underlying joint pathology may have been missed and would contribute to the long term FMPI scores.

The nature and design of the Boge et al study. (2020) means there are limitations that prevent definitive treatment recommendations being made from the findings described. The potential for bias exists due to the retrospective nature, including responder and non-responder bias, and variation in treatment between patients between clinicians over a time period. Multiple factors may have affected if a patient was managed medically or surgically, including surgeon assessment of stifle stability, presence of concomitant injuries, presence of comorbidities, demeanor of patient, degree of pain/lameness and financial factors. Some of these factors may also have affected the healing process, including degree of instability, degree of trauma to the joint, concomitant injuries, and comorbidities. Furthermore, as no diagnostic imaging or osteoarthritis scoring was performed, underlying, undiagnosed joint pathology may have affected to the healing process.

While Boge et al. (2020) adds some credence to conservative management as a treatment option of cranial cruciate disease in cats, the limitations of this study mean that further research would be required before definitive evidence-based recommendation can be made. At this time, clinicians must rely on their clinical judgement of each individual case until further evidence exists.

Gait analysis and force plate analysis are outcome measures that have been used previously in evaluating long-term outcomes in canine cranial cruciate disease (Amimoto et al., (2020)). Applying these methods to feline cranial cruciate disease could aid in a more definitive recommendation in the future.

There has been no long-term comparison of outcomes between conservative management and any other surgical procedures for the management of feline cruciate rupture e.g. TPLO. Therefore, no conclusions can be drawn on the long-term outcomes of these surgeries at this point.

Overall, the current evidence is not sufficient to draw a definitive conclusion on the long-term outcomes of surgical management of feline cranial cruciate ligament disease, compared with conservative management.

## Methodology

**Search Strategy:** 

Databases searched and dates covered	CAB Abstracts via Web of Science (1973 to Jan 2024) PubMed via the NCBI platform (1910 to Jan 2024)
Search terms	CAB Abstracts: (cat or cats or feline*).mp. (cruciate and (ligament or rupture or disease or injury)).mp. (surgical* or surger* or 'lateral suture' or 'lateral fabellotibial suture' or LFS).mp. (conservative* or medical*).mp. 1 and 2 and 3 and 4 PubMed: ((cat OR cats OR feline) AND ("cruciate ligament" or "cruciate rupture" or "cruciate disease" or "cruciate injury") AND (surgical or surgery or "lateral suture" or "lateral fabellotibial suture") AND (conservative OR medical) AND ("long term" OR "long-term" OR "outcome"))
Dates searches performed:	03 Jan 2024

Table note placeholder

**Exclusion / Inclusion Criteria:** 

Exclusion	Articles not written in English. Articles not relating to the management of isolated cranial cruciate disease in cats. No comparison between surgically and conservatively managed patients. Case reports. Case studies. Book Chapters. Conferences. Systematic reviews.
Inclusion	Meta-analysis. Randomised controlled study. Retrospective cohort.

Table note placeholder

**Search Outcome:** 

Database	Number of results	Excluded — not relating to the management of isolated cranial cruciate disease in cats	Excluded— no comparison	Excluded— single case report	Excluded— book chapter	Excluded— not written in english	Excluded — conference presentation	Total relevant papers
CAB Abstracts	18	6	2	2	1	4	2	1
PubMed	7	3	3	0	0	0	0	1
Total relevant papers when duplicates removed								1

Table note placeholder

## ORCiD

Philip Montgomery: https://orcid.org/0009-0000-1855-5643

## Conflict of Interest

The authors declare no conflicts of interest.

## Disclaimer

Knowledge Summaries are a peer-reviewed article type which aims to answer a clinical question based on the best available current evidence. It does not override the responsibility of the practitioner. Informed decisions should be made by considering such factors as individual clinical expertise and judgement along with patient’s circumstances and owners’ values. Knowledge Summaries are a resource to help inform and any opinions expressed within the Knowledge Summaries are the author’s own and do not necessarily reflect the view of the RCVS Knowledge. Authors are responsible for the accuracy of the content. While the Editor and Publisher believe that all content herein are in accord with current recommendations and practice at the time of publication, they accept no legal responsibility for any errors or omissions, and make no warranty, express or implied, with respect to material contained within. For further information please refer to our Terms of Use.
